# Exploring the Feasibility of an Examiner-Worn Neck-Mounted Camera for Objective Structured Clinical Examination Assessment: Pilot Feasibility Study

**DOI:** 10.2196/87483

**Published:** 2026-05-27

**Authors:** Miwa Sekine, Yuji Nishizaki, Amane Endo, Takasuke Ogawa, Yoshihide Takeshita, Motomi Nasu, Hiroo Wada, Chizuko Miyamoto, Michiko Oguro, Chie Otake, Yasuhiko Konishi, Yuichi Tomiki

**Affiliations:** 1 Division of Medical Education Faculty of Medicine Juntendo University Bunkyo, Tokyo Japan; 2 Chiba Faculty of Nursing Tokyo Healthcare University Funabashi City, Chiba Japan; 3 Graduate School of Nursing Science St. Luke's International University Chuo City, Tokyo Japan

**Keywords:** objective structured clinical examination, OSCE, wearable camera, medical education, assessment, feasibility study

## Abstract

**Background:**

The Objective Structured Clinical Examination (OSCE) is a prevalent method for evaluating clinical competence in medical education. As OSCEs become increasingly standardized and resource intensive, alternative evaluation methods are being explored, particularly because of the limited availability of certified examiners. However, few studies have investigated whether wearable technologies can support OSCE assessment. Wearable devices may provide a means of recording clinical skills from the examiner’s perspective.

**Objective:**

This pilot study, conducted in 2024, aimed to investigate the feasibility of using an examiner-worn neck-mounted camera for recording OSCE scenarios and to evaluate the evaluability of clinical performance from the recorded footage.

**Methods:**

In total, 9 experienced medical educators participated in a simulated OSCE scenario involving electrocardiogram lead placement. All participants completed the initial live assessment and the postuse questionnaire, while 8 of 9 (89%) participants completed the subsequent video-based reassessment. Video recordings from both a fixed camera and a neck-mounted camera (THINKLET) were used to assess the evaluability of each OSCE item. Following a washout period, evaluators reassessed the neck-mounted camera recordings by using the original checklist, while fixed-camera recordings were used to judge the evaluability of each item. Agreement between live and video-based scoring was analyzed using percent agreement and the Cohen κ coefficient. A postevaluation questionnaire captured evaluators’ experiences with the wearable device.

**Results:**

Cohen κ ranged from 0.258 to 0.913 (mean 0.67, SD 0.20). Across checklist observations, more items were judged to be evaluable in the neck-mounted camera recordings than in the fixed-camera recordings, particularly for tasks requiring observation of fine motor skills. Evaluators reported generally positive experiences with the device, although some noted issues related to audio quality, comfort, posture restriction, and limited visibility at low angles.

**Conclusions:**

Although further investigation is needed, this pilot study suggests that an examiner-worn neck-mounted camera may be a valuable supplementary assessment tool for selected OSCE tasks. Further work is needed to refine the device, standardize recording protocols, and clarify how it can best support review and verification alongside live evaluation.

## Introduction

The Objective Structured Clinical Examination (OSCE) is a globally recognized method for evaluating clinical competence in medical education [1]. According to the pyramid of clinical competence by Miller [2], OSCEs correspond to the “shows how” level of assessment, which assesses practical performance in simulated environments. In Japan, the OSCE was officially introduced in 2005 following several trial phases [3], and its role has expanded significantly in response to the transition from observation-based to hands-on clinical clerkships under the student physician system, which allows medical students to participate directly in patient care [4]. Medical students are now required to demonstrate essential clinical skills and professional attitudes before commencing clinical clerkships, with the OSCE serving as a critical evaluation tool [5].

In Western countries, medical schools in Australia and Germany have implemented various OSCE formats, often in alignment with national standards [6,7]. In Asia, Korea adopted OSCEs in 1994 despite institutional challenges [8], while Taiwan experienced significant changes following national policy shifts [9]. However, the structure and implementation of OSCEs vary considerably among institutions, including the number and duration of stations, target clinical competencies, and examiner training systems. Educational frameworks, regulatory bodies, and available human and technical resources all influence these variations.

Although the traditional OSCE provides benefits such as standardization, objectivity, and reliability [10,11], it also poses operational challenges, including significant costs, staffing demands, and logistical complexities [6]. These practical limitations have led to critical discussions concerning the feasibility and scalability of OSCEs in real-world educational contexts [12-14]. In response to these challenges, virtual OSCEs have gained prominence, particularly during the COVID-19 pandemic, necessitating the evaluation of new competencies such as “webside manner” and remote communication [15].

In 2023, Japan implemented a national certification system mandating that OSCE examiners undergo formal training and accreditation, signaling a move toward standardizing the quality of clinical skills assessment [16]. This policy reflects Japan’s effort to standardize OSCE assessment quality at the national level and illustrates the increasingly stringent requirements placed on OSCE examiners. However, the training and certification process, as required by the Common Achievement Tests Organization, is resource intensive and often impractical for faculty members who are already overburdened. Consequently, in October 2024, national guidelines were amended to allow OSCE sessions to be conducted with only 1 certified examiner per testing room, provided that the evaluation can subsequently be verified using video recordings [16].

Currently, most OSCE stations are monitored using fixed cameras. However, these systems frequently encounter limitations such as restricted angles and obstructed views, particularly when assessors or examinees block the field of view or when fine motor procedures are involved [17]. Such limitations impede accurate post hoc evaluations and may compromise the reliability of skill assessments.

Given the logistical challenges associated with securing certified examiners and the technical limitations of fixed-camera recordings, such as blind spots and obstructed views, there is an increasing need for alternative methods that can ensure reliable assessment with fewer personnel. To the best of our knowledge, no published study has evaluated the use of a neck-mounted wearable camera for OSCE assessment, and no prior research has specifically examined the THINKLET system. This study addresses this gap by investigating the feasibility of using an examiner-worn neck-mounted camera during OSCE assessments and its potential role as a supplementary tool for reviewing selected procedural tasks in settings with limited examiner availability.

## Methods

### Study Design

We undertook this pilot study to evaluate the feasibility and utility of a neck-mounted wearable camera (THINKLET, Fairy Devices Inc) for retrospective analysis in a simulated OSCE environment. Additionally, the study incorporated a questionnaire to assess the device’s usability and acceptability. The OSCE scenario focused on the placement of 12-lead electrocardiogram (ECG) electrodes, a skill frequently evaluated in OSCEs. We selected the ECG electrode placement because it requires close observation of hand positioning and electrode placement relative to anatomical landmarks, which can be difficult to capture adequately using fixed-position cameras.

### Participants

A fifth-year medical student volunteered to serve as the examinee. Nine faculty members, all with experience in clinical education, participated as evaluators. Demographic data were available for 8 evaluators; 1 evaluator did not complete the demographic questionnaire. All 9 evaluators took part in the initial live assessment; however, 1 of 9 (11%) evaluators did not participate in the subsequent video-based reassessment. We summarized continuous variables using means and SDs and presented categorical variables as frequencies and percentages.

### Scenario and Checklist Development

The research team developed the OSCE scenario and checklist in accordance with the publicly accessible guideline, “Learning and Evaluation Items for Skills and Attitudes Required for Students Participating in Clinical Clerkships,” published by the Common Achievement Tests Organization. The scenario encompassed a standard patient interaction and a procedural task related to the placement of ECG electrodes. The checklist, refined through expert consultation, comprised 26 binary items, with responses recorded as “1=performed” or “0=not performed.”

### Procedures

#### Evaluation 1 (Initial Assessment)

During the initial assessment phase, each evaluator wore a neck-mounted camera (THINKLET) while participating in the simulated OSCE. Before each session, we conducted a brief test recording to confirm the camera angle and audio functionality. Concurrent recordings from the wearable camera and a stationary camera were obtained. Assessments were conducted on October 26, 2024 (6/9 evaluators, 67%) and December 16, 2024 (3/9 evaluators, 33%), with real-time scoring using a 26-item checklist. The complete checklist items are provided in Table S1 of Multimedia Appendix 1.

#### Evaluation 2 (Video-Based Reassessment)

Following a washout period of approximately 1 month, evaluators reassessed the same OSCE performance using neck-mounted camera recordings and the original 26-item checklist. We disseminated requests for reassessment on December 2, 2024, to 6 of 9 (67%) evaluators and on January 17, 2025, to 3 of 9 (33%) evaluators. Of the 9 evaluators, 8 (89%) completed the video-based reassessment; 1 (11%) evaluator did not respond and was consequently excluded from the agreement analysis. For the fixed-camera recordings, evaluators did not rescore the performance; rather, they judged whether each checklist item was evaluable or not evaluable based on the visibility of the relevant action in the footage.

### Devices

**Figure 1:**
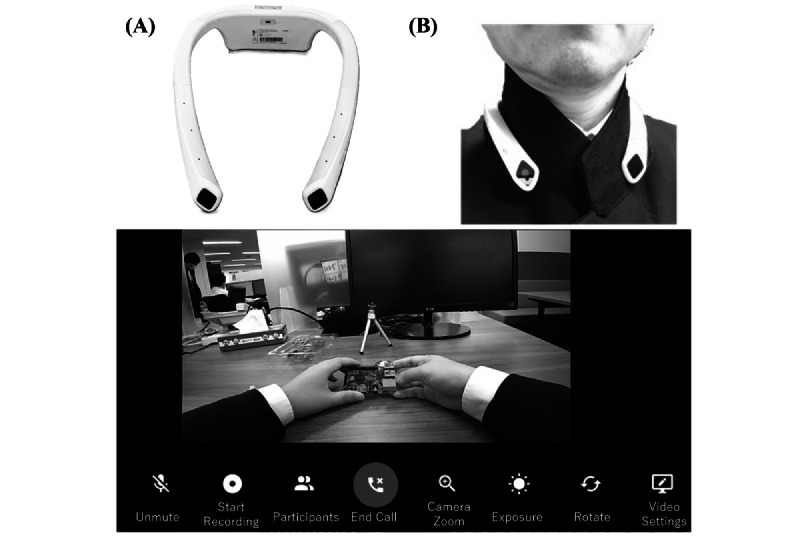
Overview of the neck-mounted wearable camera and its setup in the Objective Structured Clinical Examination environment. (A) Front view of the neck-mounted wearable camera device, (B) example of the device worn around the neck, and (C) example of a live video feed captured from the neck-mounted camera during a manual task, as displayed in an online conferencing interface.

The neck-mounted wearable camera used in this study was the THINKLET system, operated through the on-premises version of the Mirai Connect platform (Mirait One Systems Corporation). This device comprises an integrated camera and microphone, facilitating real-time communication via long term evolution or Wi-Fi. For this study, we used video and audio recordings. We recorded offline on a general-purpose PC and subsequently stored the recordings on a dedicated server. We captured audio in pulse code modulation 24-bit 48 kHz format and provided it to evaluators as complete video files. We obtained video recordings from both the fixed camera and the neck-mounted camera (refer to Multimedia Appendix 2 for an example of wearable camera footage). Figure 1 illustrates the configuration of the neck-mounted device. The device was rented from Mirait One Systems Corporation as a commercial service. The company provided technical support for device operation and troubleshooting but had no role in the study design, data collection, data analysis, interpretation of the results, or manuscript preparation.

### Questionnaire

Following the evaluations, each participant completed a questionnaire designed to assess 2 primary aspects: the comfort and usability of the wearable device during use, and the perceived ease and effectiveness of evaluating student performance using footage from wearable devices rather than fixed-camera footage. The questionnaire comprised 8 closed-ended items rated on a 5-point Likert scale, along with 2 open-ended questions. The questionnaire items on device usability and video-based evaluation are provided in Tables S2 and S3 of Multimedia Appendix 1.

### Data Analysis

#### Descriptive Statistics

We undertook a descriptive analysis of the demographic data and the evaluators’ backgrounds. We reported means and SDs for continuous variables and presented categorical variables as frequencies and percentages.

#### Agreement Between Live and Video-Based Scoring

We analyzed agreement between evaluation 1 and the video-based reassessment using percentage agreement and Cohen κ coefficient [18] and excluded items designated as “not evaluable” or missing data. We interpreted κ values in accordance with guidelines established by Landis and Koch [19].

### Evaluability by Camera Type

For each checklist item, we descriptively compared evaluability (“evaluable” vs “not evaluable”) between recordings from a fixed camera and those from a neck-mounted camera. Results are presented as counts and percentages of evaluable and nonevaluable observations for each camera type.

### Questionnaire Analysis

We summarized responses to the Likert-scale items by calculating frequencies and percentages. We used thematic coding to analyze free-text responses, facilitating the identification of recurring content categories. Furthermore, we manually applied a basic sentiment classification, categorized as positive, negative, or neutral, to each response based on its overall tone. We selected this manual approach to ensure contextual accuracy and minimize the risk of misclassification due to nuanced expressions or cultural subtleties that automated sentiment analysis tools frequently overlook.

### Software

We performed data analyses using Microsoft Excel and R (version 4.3.3; R Foundation for Statistical Computing).

### Ethical Considerations

The Juntendo University Ethical Review Board (approval number E24-0093) approved this study. The study followed the Strengthening the Reporting of Observational Studies in Epidemiology reporting guidelines. All participants, including the student examinee and faculty evaluators, provided written informed consent before participation, including consent for video recording and publication of selected materials. The student examinee received compensation for participation, whereas the faculty evaluators did not receive financial compensation. Because the study involved video and audio recordings that could contain identifiable information, the data were stored on a secure institutional server with access restricted to the research team. Identifiable information, such as faces and name badges, was masked in publication materials when appropriate. A selected video excerpt was submitted for publication.

## Results

### Participant Characteristics

A total of 9 evaluators participated. Demographic data were available for 8 of 9 (89%) evaluators, as 1 of 9 (11%) evaluators did not complete the demographic questionnaire. Among these 8 evaluators, 7 (88%) were men and 1 (13%) was a woman, with a mean age of 42.1 (SD 8.9) years and a mean of 14.9 years (SD 9.1) since graduation. All respondents had graduated from a medical school. Overall, 3 (38%) evaluators had prior experience as officially certified OSCE examiners, having completed the formal national accreditation process, while the remaining 5 (63%) had no such certification. In total, 5 (56%) evaluators had never previously participated in an OSCE evaluation, whereas 3 (33%) had done so ≥5 times.

### Agreement Between Live and Video-Based Scoring

#### Item-Based Agreement

Among the 26 checklist items, most demonstrated high concordance (75% to 100%) between the initial live assessments and video-based reassessments. Three items—removal of clothing (item 4), placement of chest lead C2 (item 12), and postprocedure tidying up (item 25)—achieved 100% agreement. In contrast, item 20, communication during the examination: instructing the patient to relax, had the lowest agreement at 50%. Table S4 in Multimedia Appendix 1 provides detailed item-level results.

#### Evaluator-Based Agreement

The level of agreement between each evaluator’s initial and reassessment scores varied, with percentage agreement ranging from 70% to 96% and Cohen κ coefficient ranging from 0.258 to 0.913. The mean κ across all evaluators was 0.67 (SD 0.20). One evaluator showed only fair agreement (Cohen κ=0.26). Table S5 in Multimedia Appendix 1 provides detailed evaluator-specific results.

### Evaluability by Camera Type

**Figure 2:**
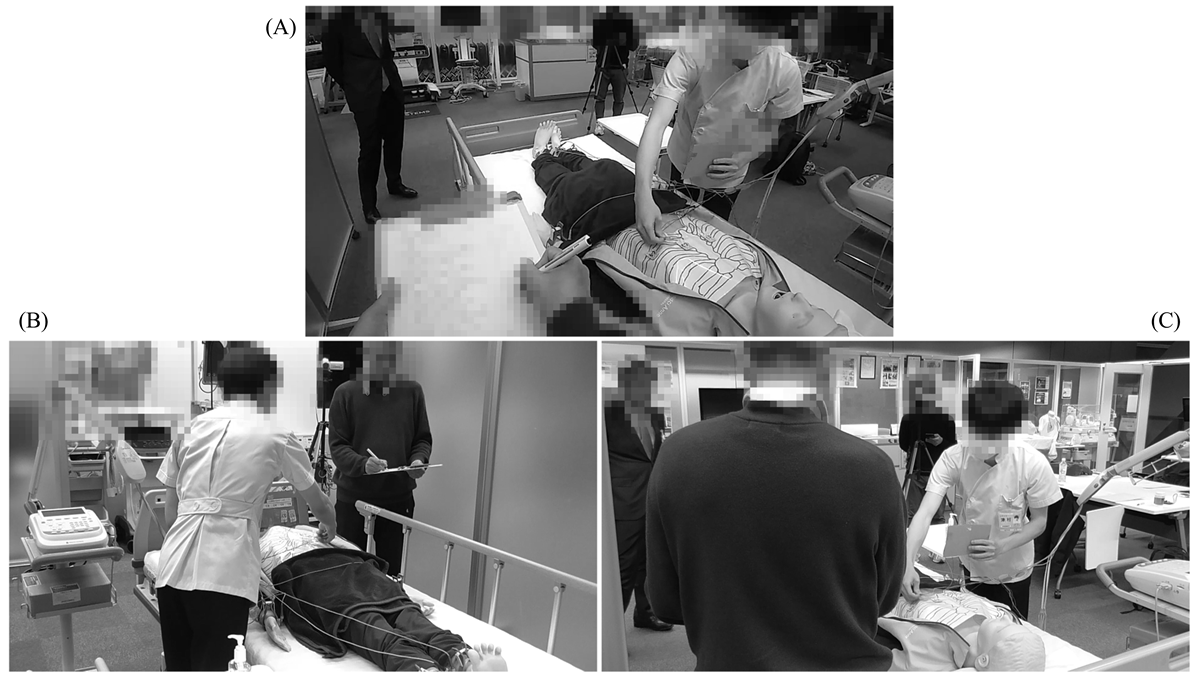
Comparison of camera perspectives during an Objective Structured Clinical Examination station on 12-lead electrocardiogram electrode placement. (A) View captured from the neck-mounted camera worn by the examiner, (B) view captured from a foot-side fixed camera, and (C) view captured from a head-side fixed camera.

Figure 2 presents visual examples of the perspectives captured by the neck-mounted and fixed cameras. In the fixed-camera recordings, some tasks requiring fine motor skills, such as electrode placements at C5 and C6, were more frequently judged as not evaluable than in the neck-mounted camera recordings (Table 1). Across all checklist observations, 206 were judged evaluable in the neck-mounted camera recordings, compared with 185 in the fixed-camera recordings (Table 2). Several electrode placement tasks, including C5 and C6 placement, were more frequently judged evaluable in the neck-mounted camera recordings than in the fixed-camera recordings (Table 1). Multimedia Appendix 2 provides an example of this type of footage.

**Table 1:** Comparison of evaluability: fixed vs neck-mounted camera.

Objective Structured Clinical Examination checklist item		Evaluation feasibility, n (%)	
		Fixed camera (n=8)	Neck-mounted camera (n=8)
**Patient confirmation and preparation**			
	Hand hygiene: disinfect hands	8 (100)	8 (100)
	Patient identification: confirm the patient’s identity using full name and date of birth	8 (100)	8 (100)
	Explanation of procedure: briefly explain the purpose and process of the ECG^a^ to the patient	8 (100)	8 (100)
	Clothing removal: ask the patient to remove upper-body garments, socks, and stockings	8 (100)	8 (100)
	Privacy protection: provide appropriate coverage using a towel	8 (100)	8 (100)
**Preparation of ECG equipment and environment**			
	Environmental check: ensure no other electrical devices are within 10 feet of the ECG machine	8 (100)	8 (100)
	Device check: turn on the ECG device and confirm that it is functioning properly	8 (100)	8 (100)
**Electrode placement**			
	Order of attachment: attach limb electrodes first, followed by precordial electrodes	7 (100)^b^	8 (100)
	Limb lead placement: attach electrodes to the palmar side of the wrists and medial side of the ankles	7 (87.5)	8 (100)
	Precordial lead preparation: palpate the sternal angle and identify the second intercostal space	7 (87.5)	8 (100)
	Precordial lead placement: place C1 at the fourth intercostal space, right sternal border	7 (87.5)	8 (100)
	Precordial lead placement: place C2 at the fourth intercostal space, left sternal border	7 (87.5)	8 (100)
	Precordial lead placement: place C4 at the intersection of the fifth intercostal space and midclavicular line	6 (75)	8 (100)
	Precordial lead placement: place C3 midway between C2 and C4	6 (75)	8 (100)
	Precordial lead placement: place C5 at the same level as C4 on the left anterior axillary line	4 (50)	7 (87.5)
	Precordial lead placement: place C6 at the same level as C4 on the left midaxillary line	4 (50)	7 (87.5)
	Precordial lead placement: confirm the correct order from C1 to C6	6 (75)	8 (100)
	Precordial lead placement: ensure that adjacent electrodes do not touch each other	7 (87.5)	8 (100)
**Procedure and communication**			
	Communication during attachment: inform the patient before attaching the electrodes	8 (100)	8 (100)
	Communication during measurement: encourage the patient to relax and remain still	8 (100)	8 (100)
**Postprocedure handling**			
	Electrode removal: carefully remove electrodes to avoid skin irritation	7 (100)^b^	8 (100)
	End-of-test communication: notify the patient that the measurement is complete before removing the electrodes	8 (100)	8 (100)
	Electrode removal order: first remove the precordial electrodes, then the limb electrodes	8 (100)	8 (100)
	Electrode removal order: remove the right leg (ground) electrode last	6 (75)	8 (100)
	Cleanup: prepare electrodes and related items appropriately for next use	8 (100)	8 (100)
**Closure**			
	Completion communication: inform the patient that the test is complete and instruct them to get dressed	8 (100)	8 (100)

^a^ECG: electrocardiogram.

^b^n=7 evaluators.

**Table 2:** Descriptive comparison of item evaluability between fixed camera and examiner-worn neck-mounted camera recordings^a^.

Category	Fixed camera			Neck-mounted camera	
	Evaluable (n=185), n (%)	Not evaluable (n=21), n (%)	Evaluable (n=206), n (%)		Not evaluable (n=2), n (%)
Patient confirmation and preparation	40 (21.6)	0 (0)	40 (19.4)		0 (0)
Preparation of electrocardiogram equipment and environment	16 (8.6)	0 (0)	16 (7.8)		0 (0)
Electrode placement	68 (36.8)	19 (90.5)	86 (41.7)		2 (100)
Procedure and communication	16 (8.6)	0 (0)	16 (7.8)		0 (0)
Postprocedure handling	37 (20)	2 (9.5)	40 (19.4)		0 (0)
Closure	8 (4.3)	0 (0)	8 (3.9)		0 (0)

^a^The table presents a descriptive comparison of evaluability between the fixed camera and the neck-mounted camera, based on the number of evaluable and not evaluable observations in each category.

### Questionnaire Results

In the context of video-based evaluation using wearable camera footage, 7 (88%) of 8 evaluators rewound and rewatched the videos during assessment. In total, 3 (38%) of 8 evaluators reported difficulty hearing audio, and 3 (38%) reported difficulty observing the desired area. Half (n=4, 50%) of the evaluators agreed, and 1 (13%) strongly agreed, that video quality was equivalent to that of on-site evaluation. Most (n=7, 88%) evaluators strongly agreed or agreed that video-based evaluation should be integrated into OSCE assessment. Tables S6 and S7 in Multimedia Appendix 1 provide detailed results, and Table 3 shows a summary of free-text feedback.

**Table 3:** Summary of free-text feedback.

Category and feedback			Coded comments, n		Representative comments
**Usability and comfort**					
	Positive	11		“I was initially worried the device might fall off, but I gradually got used to it.” “I didn’t feel any burden from the weight, which was good.” “I was able to use it without any discomfort.”	
	Negative	4		“A bit hot.” “The back of my head felt warm.” “It might feel slightly hot in summer if worn for a long time.” “I felt heat around my neck.”	
**Technical issues and attachment**					
	Positive	1		“Honestly, I was surprised by the high quality of the wearable device.”	
	Negative	13		“It would be good if the position could be fixed. If it slips, I worry the camera will show what’s below the body.” “I felt I needed to be aware that the camera’s view might differ from what I was actually seeing.” “How to hold the evaluation sheet needs consideration.” “It was hard to decide where to put the binder.” “I was concerned the evaluation sheet might appear on screen.” “Though the device worked during the check, during the evaluation, it only showed my neck and glasses.” “Depending on how its worn, wearable devices may not capture low-positioned items.” “Familiarity may help, but placing the evaluation form was difficult.” “It was difficult to write on the form, which delayed evaluation.” “Audio cuts out.” “Audio issues.” “I was somewhat conscious about what the camera was capturing, but it didn’t affect my evaluation.” “I was worried the evaluation notebook might be visible.”	
**Benefits of video-based evaluation**					
	Positive	8		“I could evaluate just like in a real-time situation.” “Using this device could reduce the burden on OSCE evaluators.” “Fixed cameras can’t capture hands, but wearables can.” “Replaying improves evaluation accuracy.”	
	Negative	0		None	

The evaluators’ positive comments commonly noted good usability and lack of discomfort, while their negative comments frequently mentioned heat sensation, device slippage, and difficulty handling the evaluation sheet. Counts represent the number of coded comments derived from the free-text responses.

## Discussion

### Principal Findings

This study investigated the feasibility of using an examiner-worn neck-mounted camera for OSCE assessment. Agreement between live and video-based scoring varied across evaluators, with Cohen κ values ranging from 0.258 to 0.913. In the descriptive comparison of camera perspectives, more checklist items were judged evaluable in neck-mounted camera recordings than in fixed-camera recordings, particularly for selected electrode placement tasks requiring fine motor observation. Evaluator feedback indicated both perceived usefulness and practical limitations. Although many evaluators supported incorporating video-based assessment into OSCE practice, others reported technical challenges, including audio issues, visibility of the target area, heat sensation, device slippage, and difficulty handling the evaluation sheet (Table 3). Together, these findings suggest that an examiner-worn neck-mounted camera may be useful as a supplementary tool for review and verification in selected OSCE tasks, rather than as a replacement for live assessment. Further refinement of device setup and recording conditions is needed before broader implementation.

This study directly compared live and video-based OSCE assessments using neck-mounted and fixed-camera recordings in a simulated educational scenario. Our methodology provided descriptive information regarding the evaluability of individual checklist items. We also gathered and reported evaluator background information, thereby enhancing the transparency of the study context. Furthermore, we incorporated both quantitative and qualitative feedback on device usability to support the interpretation of the feasibility findings.

The COVID-19 pandemic, which began in 2019, prompted a significant shift toward remote and online methods across various sectors, including medical education [20]. As telemedicine, online learning, and virtual assessments proliferated [21,22], the adoption of wearable technologies also gained traction [22].

Wearable devices have garnered increasing attention as instruments for clinical education and performance evaluation [23]. The expansion of remote and hybrid teaching during the COVID-19 pandemic further accelerated interest in their application for clinical skills training. These devices provide a first-person perspective, offering wearable camera recordings that facilitate detailed observation of fine motor skills and examinee behaviors [24,25]. In addition to passive recording, certain devices also support real-time, bidirectional communication, enabling remote instructors or supervisors to deliver immediate feedback during clinical tasks. This interactive capability further enhances their utility in remote education and simulation-based training environments. In online education, these recordings have been associated with increased student satisfaction and perceived educational effectiveness [23,26]. These characteristics may make wearable cameras a potentially relevant option for examiner-perspective recording in OSCE assessment.

Although wearable devices offer distinct advantages, they may not be suitable for all assessment domains; for example, fixed third-person cameras may be more appropriate for evaluating nonverbal communication [25]. Wearable and related observational technologies have been explored in health research, OSCE-related contexts, and other applied settings [27-31]. In this study, we focused specifically on the potential role of an examiner-worn neck-mounted camera in OSCE assessment.

Although various wearable devices, including smart glasses and action cameras, have been used globally to evaluate communication with standardized patients and develop instructional content [32,33], they are not without limitations. Reported challenges encompass device weight, image instability, limited field of view, and privacy concerns [34,35]. Smart glasses have been associated with visual distraction and diminished attentional focus because of their design, including a limited field of view and camera instability. These limitations may impede clear observation of procedural tasks and compromise the reliability of recorded footage, particularly in high-stakes assessment settings such as OSCEs [36-38]. These challenges indicate the importance of ergonomic and design considerations in wearable camera systems. These considerations are particularly relevant to the use of examiner-worn cameras in OSCE assessment, where stable first-person recording of procedural tasks is required. Research has identified smart glasses as less user-friendly regarding operability and comfort [36], which influenced our decision to implement a neck-mounted camera design. In this pilot study, evaluator feedback suggested that the neck-mounted camera was generally acceptable to some users and may offer practical advantages for reviewing selected procedural tasks.

Nonetheless, we observed some technical limitations. For example, visibility was occasionally obstructed when evaluators held the checklist close at hand, which sometimes blocked the camera’s view. In addition, there were challenges related to audio capture because the recording software did not allow remote control of the microphone’s on-off function due to privacy regulations. Consequently, evaluators could not easily confirm whether audio was being recorded, which occasionally made it difficult to ensure adequate audio quality for precise assessment.

Furthermore, we noted variability in interrater agreement (Cohen κ=0.258-0.913), potentially influenced by inconsistencies in video framing. In certain instances, key procedural steps were not clearly visible, and evaluators reported misaligned camera angles. These issues likely resulted from variations in how the device was worn, as factors such as shirt collars, ties, or fabric thickness affected the camera’s orientation and stability.

To address these challenges, future efforts should focus on standardizing attachment protocols, introducing supplementary supports to minimize clothing-related interference, enhancing microphone sensitivity, and providing user interfaces that indicate audio recording status. Conducting test recordings before actual assessments may also help ensure optimal video framing and sound quality. Moreover, broader implementation will require the establishment of standardized OSCE video-recording protocols, structured evaluator training programs, and secure data governance frameworks that comply with relevant ethical and legal standards.

### Limitations

This pilot study had some limitations. First, the small sample size and limited number of evaluators constrained the generalizability of the findings. Additionally, the examinee was a fifth-year medical student participating in a mock OSCE, which may not fully replicate actual examination conditions. Second, the evaluators were predominantly faculty members from a single institution, with only 1 evaluator from another university, thereby limiting the diversity of perspectives. Third, the study focused exclusively on a single OSCE scenario, ECG electrode placement, rendering it challenging to extrapolate the findings to other clinical skills or specialties. Additionally, this study did not evaluate scoring accuracy against an external reference standard; therefore, the findings should not be interpreted as evidence that neck-mounted recordings improve assessment accuracy. Furthermore, agreement analyses were based on reassessment using the neck-mounted recordings, whereas fixed-camera recordings were used only to judge whether checklist items were evaluable. Technical issues, including audio quality, visibility, and device positioning, also limited the consistency of video-based reassessment. Future research involving larger sample sizes, multi-institutional collaboration, and multiple OSCE stations is necessary to validate the broader applicability of neck-mounted cameras.

### Conclusions

This pilot study investigated the feasibility of using an examiner-worn neck-mounted camera in preclinical OSCE assessments. A greater number of checklist items were judged evaluable in neck-mounted camera recordings than in fixed-camera recordings, particularly for selected tasks requiring fine motor observation. These findings suggest that an examiner-worn neck-mounted camera may be useful as a supplementary tool for review and verification in selected OSCE tasks rather than as a replacement for live assessment. However, technical and operational challenges remain, indicating the need for further refinement of device design and evaluation protocols in medical education before broader implementation.

## Data Availability

The datasets generated and analyzed during this study, including video recordings featuring participants, are not publicly available due to ethical restrictions and participant confidentiality. However, they are available from the corresponding author upon reasonable request and with approval from the institutional ethics committee.

## Appendix

Multimedia Appendix 1 Detailed Objective Structured Clinical Examination checklist, interrater agreement, and questionnaire items or responses used in the study.

Multimedia Appendix 2 A 60-second excerpt from a neck-mounted camera (THINKLET, Fairy Devices Inc) worn by the examiner during a simulated Objective Structured Clinical Examination station on 12-lead electrocardiogram electrode placement.

## Abbreviations

ECG: electrocardiogram
OSCE: Objective Structured Clinical Examination
