# Supplementary material for: Exploring the Feasibility of an Examiner-Worn Neck-Mounted Camera for Objective Structured Clinical Examination Assessment: Pilot Feasibility Study
Source: JMIR Med Educ. 2026 May 27;12:e87483. doi: 10.2196/87483 (PMC13254511; doi:10.2196/87483)
Supplement: Multimedia Appendix 1 [file mededu_v12i1e87483_app1.docx]

**Table S1. ECG OSCE Checklist with Interrater Agreement**

| **Evaluation Category** | **Item No.** | **Checklist Item** |
| --- | --- | --- |
| 1. Patient confirmation and preparation | 1 | Hand hygiene: Disinfect hands. |
|  | 2 | Patient identification: Confirm the patient’s identity using full name and date of birth. |
|  | 3 | Explanation of procedure: Briefly explain the purpose and process of the ECG to the patient. |
|  | 4 | Clothing removal: Ask the patient to remove upper-body garments, socks, and stockings. |
|  | 5 | Privacy protection: Provide appropriate coverage using a towel. |
| 2. Preparation of ECG equipment and environment | 6 | Environmental check: Ensure that no other electrical devices are near the ECG machine. |
|  | 7 | Device check: Turn on the ECG device and confirm that it is functioning properly. |
| 3. Electrode placement | 8 | Order of attachment: Attach limb electrodes first, followed by precordial electrodes. |
|  | 9 | Limb lead placement: Attach electrodes to the palmar side of the wrists and medial side of the ankles. |
|  | 10 | Precordial lead preparation: Palpate the sternal angle and identify the second intercostal space. |
|  | 11 | Precordial lead placement: Place C1 at the 4th intercostal space, right sternal border. |
|  | 12 | Precordial lead placement: Place C2 at the 4th intercostal space, left sternal border. |
|  | 13 | Precordial lead placement: Place C4 at the intersection of the 5th intercostal space and midclavicular line. |
|  | 14 | Precordial lead placement: Place C3 midway between C2 and C4. |
|  | 15 | Precordial lead placement: Place C5 at the same level as C4 on the left anterior axillary line. |
|  | 16 | Precordial lead placement: Place C6 at the same level as C4 on the left midaxillary line. |
|  | 17 | Precordial lead placement: Confirm the correct order from C1 to C6. |
|  | 18 | Precordial lead placement: Ensure that adjacent electrodes do not touch each other. |
| 4. Procedure and communication | 19 | Communication during attachment: Inform the patient prior to attaching the electrodes. |
|  | 20 | Communication during measurement: Encourage the patient to relax and remain still. |
| 5. Post-procedure handling | 21 | Electrode removal: Carefully remove electrodes to avoid skin irritation. |
|  | 22 | End-of-test communication: Notify the patient that the measurement is complete before removing electrodes. |
|  | 23 | Electrode removal order: First remove the precordial electrodes, then the limb electrodes. |
|  | 24 | Electrode removal order: Remove the right leg (ground) electrode last. |
|  | 25 | Clean-up: Prepare electrodes and related items appropriately for next use. |
| 6. Closure | 26 | Completion communication: Inform the patient that the test is complete and instruct them to get dressed. |

**Table S2. Perceptions of Wearing a Neck-Mounted Wearable Device**

| 1. Did you feel restricted in your movements while wearing the device? |
| --- |
| Strongly Disagree |
| Disagree |
| Neutral |
| Agree |
| Strongly Agree |
| 2. Did you feel restricted in your posture while wearing the device? |
| Strongly Disagree |
| Disagree |
| Neutral |
| Agree |
| Strongly Agree |
| 3. Did the weight of the device feel burdensome? |
| Strongly Disagree |
| Disagree |
| Neutral |
| Agree |
| Strongly Agree |
| 4. Did the fit of the device feel burdensome? |
| Strongly Disagree |
| Disagree |
| Neutral |
| Agree |
| Strongly Agree |
| 5. Did wearing the device make it difficult to concentrate on the assessment task? |
| Strongly Disagree |
| Disagree |
| Neutral |
| Agree |
| Strongly Agree |

**Table S3. Evaluation Using Neck-Mounted Wearable Camera Footage**

| Did you rewind and rewatch the videos during evaluation? |
| --- |
| No |
| Yes |
| Was it difficult to hear the audio during the video-based evaluation? |
| Strongly disagree |
| Disagree |
| Neutral |
| Agree |
| Strongly agree |
| Was it difficult to see the area you wanted to observe in the video? |
| Strongly disagree |
| Disagree |
| Neutral |
| Agree |
| Strongly agree |
| Compared to on-site evaluation, did the video maintain equivalent quality? |
| Strongly disagree |
| Disagree |
| Neutral |
| Agree |
| Strongly agree |
| Do you think video-based evaluation should be integrated into OSCE assessment? |
| Strongly disagree |
| Disagree |
| Neutral |
| Agree |
| Strongly agree |

# Table S4. ECG OSCE Checklist with Interrater Agreement

| **Evaluation Category** | **Item No.** | **Checklist Item** | **Interrater Agreement** |
| --- | --- | --- | --- |
| 1. Patient confirmation and preparation | 1 | Hand hygiene: Disinfect hands. | 75%  (6/8) |
|  | 2 | Patient identification: Confirm the patient’s identity using full name and date of birth. | 87.5% (7/8) |
|  | 3 | Explanation of procedure: Briefly explain the purpose and process of the ECG to the patient. | 87.5% (7/8) |
|  | 4 | Clothing removal: Ask the patient to remove upper-body garments, socks, and stockings. | 100% (8/8) |
|  | 5 | Privacy protection: Provide appropriate coverage using a towel. | 87.5% (7/8) |
| 2. Preparation of ECG equipment and environment | 6 | Environmental check: Ensure that no other electrical devices are near the ECG machine. | 87.5% (7/8) |
|  | 7 | Device check: Turn on the ECG device and confirm that it is functioning properly. | 75%  (6/8) |
| 3. Electrode placement | 8 | Order of attachment: Attach limb electrodes first, followed by precordial electrodes. | 87.5% (7/8) |
|  | 9 | Limb lead placement: Attach electrodes to the palmar side of the wrists and medial side of the ankles. | 87.5% (7/8) |
|  | 10 | Precordial lead preparation: Palpate the sternal angle and identify the second intercostal space. | 75%  (6/8) |
|  | 11 | Precordial lead placement: Place C1 at the 4th intercostal space, right sternal border. | 87.5% (7/8) |
|  | 12 | Precordial lead placement: Place C2 at the 4th intercostal space, left sternal border. | 100% (8/8) |
|  | 13 | Precordial lead placement: Place C4 at the intersection of the 5th intercostal space and midclavicular line. | 87.5% (7/8) |
|  | 14 | Precordial lead placement: Place C3 midway between C2 and C4. | 75%  (6/8) |
|  | 15 | Precordial lead placement: Place C5 at the same level as C4 on the left anterior axillary line. | 87.5% (7/8) |
|  | 16 | Precordial lead placement: Place C6 at the same level as C4 on the left midaxillary line. | 87.5% (7/8) |
|  | 17 | Precordial lead placement: Confirm the correct order from C1 to C6. | 87.5% (7/8) |
|  | 18 | Precordial lead placement: Ensure that adjacent electrodes do not touch each other. | 87.5% (7/8) |
| 4. Procedure and communication | 19 | Communication during attachment: Inform the patient prior to attaching the electrodes. | 87.5% (7/8) |
|  | 20 | Communication during measurement: Encourage the patient to relax and remain still. | 50%  (4/8) |
| 5. Post-procedure handling | 21 | Electrode removal: Carefully remove electrodes to avoid skin irritation. | 87.5% (7/8) |
|  | 22 | End-of-test communication: Notify the patient that the measurement is complete before removing electrodes. | 87.5% (7/8) |
|  | 23 | Electrode removal order: First remove the precordial electrodes, then the limb electrodes. | 87.5% (7/8) |
|  | 24 | Electrode removal order: Remove the right leg (ground) electrode last. | 75%  (6/8) |
|  | 25 | Clean-up: Prepare electrodes and related items appropriately for next use. | 100% (8/8) |
| 6. Closure | 26 | Completion communication: Inform the patient that the test is complete and instruct them to get dressed. | 75%  (6/8) |

# Table S5. Interrater Agreement for Each Evaluator

| **Evaluator** | **Agreement Rate** | **Cohen’s κ** | **Notes** |
| --- | --- | --- | --- |
| Evaluator 1 | 92% (23/25) | 0.702 | 1st round: 1 item unchecked |
| Evaluator 2 | 96.2% (25/26) | 0.913 |  |
| Evaluator 3 | 73.1% (19/26) | 0.435 |  |
| Evaluator 4 | 95.5% (21/22) | 0.776 | 1st round: 2 items unchecked, 2 items unevaluable owing to device setup issue ※ |
| Evaluator 5 | 92.3% (24/26) | 0.708 |  |
| Evaluator 6 | 92.3% (24/26) | 0.805 |  |
| Evaluator 7 | 69.6% (16/23) | 0.258 | 1st round: 3 items unchecked |
| Evaluator 8 | 92.3% (24/26) | 0.752 |  |

※Unevaluable owing to evaluator’s improper device attachment during the second-round (video-based) assessment (self-reported).

# Table S6. Perceptions of Wearing a Neck-Mounted Wearable Device (N = 9)

|  |  | N (%) |
| --- | --- | --- |
| **1. Did you feel restricted in your movements while wearing the device?** | | |
|  | Strongly disagree | 4 (44.4) |
|  | Disagree | 2 (22.2) |
|  | Neutral | 2 (22.2) |
|  | Agree | 1 (11.1) |
|  | Strongly agree | 0 (0) |
| **2. Did you feel restricted in your posture while wearing the device?** | | |
|  | Strongly disagree | 3 (33.3) |
|  | Disagree | 3 (33.3) |
|  | Neutral | 0 (0) |
|  | Agree | 3 (33.3) |
|  | Strongly agree | 0 (0) |
| **3. Did the weight of the device feel burdensome?** | |  |
|  | Strongly disagree | 7 (77.8) |
|  | Disagree | 2 (22.2) |
|  | Neutral | 0 (0) |
|  | Agree | 0 (0) |
|  | Strongly agree | 0 (0) |
| **4. Did the fit of the device feel burdensome?** | |  |
|  | Strongly disagree | 6 (66.7) |
|  | Disagree | 3 (33.3) |
|  | Neutral | 0 (0) |
|  | Agree | 0 (0) |
|  | Strongly agree | 0 (0) |
| **5. Did wearing the device make it difficult to concentrate on the assessment task?** | | |
|  | Strongly disagree | 1 (11.1) |
|  | Disagree | 7 (77.8) |
|  | Neutral | 0 (0) |
|  | Agree | 1 (11.1) |
|  | Strongly agree | 0 (0) |

# Table S7. Evaluation Using Neck-Mounted Wearable Camera Footage

|  |  | N (%) |
| --- | --- | --- |
|  | Total | 8 (100) |
| **Did you rewind and rewatch the videos during evaluation?** | |  |
|  | No | 1 (12.5) |
|  | Yes | 7 (87.5) |
| **Was it difficult to hear the audio during the video-based evaluation?** | | |
|  | Strongly disagree | 2 (25) |
|  | Disagree | 1 (12.5) |
|  | Neutral | 2 (25) |
|  | Agree | 3 (37.5) |
|  | Strongly agree | 0 (0) |
| **Was it difficult to see the area you wanted to observe in the video?** | | |
|  | Strongly disagree | 3 (37.5) |
|  | Disagree | 1 (12.5) |
|  | Neutral | 1 (12.5) |
|  | Agree | 3 (37.5) |
|  | Strongly agree | 0 (0) |
| **Compared to on-site evaluation, did the video maintain equivalent quality?** | | |
|  | Strongly disagree | 0 (0) |
|  | Disagree | 1 (12.5) |
|  | Neutral | 2 (25) |
|  | Agree | 4 (50) |
|  | Strongly agree | 1 (12.5) |
| **Do you think video-based evaluation should be integrated into OSCE assessment?** | | |
|  | Strongly disagree | 0 (0) |
|  | Disagree | 0 (0) |
|  | Neutral | 1 (12.5) |
|  | Agree | 1 (12.5) |
|  | Strongly agree | 6 (75) |
